# Arthritis, or Gout

**Published:** 1838-03-28

**Authors:** N. Chapman

**Affiliations:** Professor of the Theory and Practice of Physic, in the University of Pennsylvania


### MEDICAL EXAMINER.

DEVOTED TO MEDICINE, SURGERY AND THE COLLATERAL SCIENCES.

**EDITED by J. B. BIDDLE, M. D. and M. CLYMER, M. D.**

No. 7. PHILADELPHIA, WEDNESDAY, MARCH 28, 1838. Vol. 1.

### ARTHRITIS, OR GOUT.

*By* N. Chapman, M. D., *Professor of the Theory and Practice of Physic, in the University of Pennsylvania.*

(Continued from page 92.)

Taking the whole of what I have said into consideration, upon this subject, I think we are entitled to conclude, that purgatives may be safely and efficaciously employed, and, that they have hitherto been strangely and inconsiderately neglected.

These being so beneficial in gout, it is reasonable to suppose, from analogy, that emetics might also be so. In the practice of antiquity, as well as of more recent times, they were, indeed, made a part of the treatment, and came to be excluded, with the other evacuants, pretty much through the influence of Sydenham. The most conspicuous of those, by whom it is commended, are Celsus, Cœlius, Aurelianus, Demetrius, and several of the Arabian school, to which may be added, Fabricius, Heldanus, Gesnen, Stoll and Scudamore. No one, however, seems so strenously to have endeavored the revival of the practice, as Small, the author of a very good paper on gout.* He urges it as affording very prompt relief of the pain and inflammation of the paroxysm. Considering the nature of the case, such an effect is probable, though, I confess, my experience is not sufficiently extensive, to lead to any confirmation of its utility. Emetics, at all times, are much resisted, especially in the higher classes of society, where chiefly we meet with the disease, and this, and not any apprehension of mischief, has prevented my using them. There are cases, however, as where the stomach is greatly disordered, and the tongue loaded, in which I have prescribed them, and with advantage.

Gout, moreover, may be acquired in miasmatic districts, and, when connected with intermittent fever, and a very oppressed state of the stomach, emetics, I have found, in some instances, indispensable to the cure.

Estimating, as I do, evacuations of the alimentary canal, and particularly by purging, in gout, I have seen too much of the practice of my profession, to confide in any one curative process, and, especially, in a disease which presents so many diversities. As in all other instances, the remedies here are to be accommodated to circumstances, and, hence, in the management of this disease, I sometimes call into requisition, in its different stages or forms, every variety of treatment. It will be found necessary to resort to the lancet, where it is indicated by a strong febrile pulse or irregular determination of blood, or to relieve the intense local phlogosis, and such conditions existing, it should precede the use of evacuants of the alimentary canal, as well as all other measures.

Cases have come under my care, in which I have bled very freely, and with decisive utility. But these do not ordinarily happen, and, since I have relied so much on evacuations of the bowels, the necessity of the lancet, I have found to be infinitely less. Not the least of the great advantages of purging in this disease, is, that the full pulse, increased temperature, and other febrile, as well as the topical affections, are speedily removed. It has been remarked, too, very generally, that venesection is not productive of as much benefit in gout, as in other phlegmasia, and which I believe to be true. Nevertheless, it is of such importance, as to be even indispensable under the circumstances previously stated, and, though condemned by some of the French writers, is abundantly sustained by a host of the very highest of our authorities, from the earliest period down to the present day. No other foundation than an idle prejudice, has the objection to the practice on account of its tendency to induce retrocessions of the disease. Frequently as I have employed it, none such, at least, have I observed, and in this opinion, I know that I am well supported.

Entertaining the conviction, that it is one of those diseases, dependent on morbific matter, and that the skin is the natural emunctory for its discharge, many of the disciples of the humeral pathology indulged in the very free use of diaphorectics in gout. As subordinate means, these, unquestionably, are sometimes of service. Nature, whose intimations ought always to be consulted, and which most generally may be trusted, distinctly points to their use. The paroxysms of regular arthritis, when spontaneously cured, go off most commonly, with diarrhœa, diaphoresis, or diuresis. But there is a time, or state of the disease, in which diaphoretics are proper only. It is, after the reduction of the force of an acute attack, or earlier in those atonic cases arising out of a naturally feeble frame, or one broken down by a course of vicious indulgences. The cordial and stimulating articles are mostly to

*Medical Observer, vol. 1.

be preferred. Combinations of ammonia, either the carbonate or acetate, with laudanum, and wine whey, I have found to be admirably adapted. The Dover's powders also, where the stomach is not irritable, may sometimes be employed advantageously.

Discharges of urine, as just remarked, are often critical in this disease. This natural proclivity being, therefore, evinced, it should be promoted. The dulcified spirits of nitre are well suited to the occasion, and I have witnessed in some instances, signal utility from a combination of the tincture of colchicum with it. The urinary and perspirating processes are productive of nearly the same effect, in the cure of many diseases, and the one or the other should be encouraged, as nature seems to point out.

Completed, as I have now done, the regular treatment of gout, I must now say a word or two of a nostrum, of too much repute in this disease, to be overlooked. My allusion is to the *Eau Medicinale*, an article originally invented in France, which, at one time, had acquired immense celebrity throughout Europe. It has also crept partially into use in this country, and I have had several opportunities of witnessing its astonishing powers in the different shapes of arthritic affections. Of its composition, we have ascertained nothing with certainty, though there are various conjectures on the subject, the most probable of which is, that it is essentially colchicum. Its effects on the system are distinctly marked. As a purgative, it operates actively, and sometimes vomits violently, producing, at the same time, a copious perspiration, or diuresis, with extreme nervous distress and prostration of muscular power. During the operation, however, the pain and swelling of the joints so rapidly subside, that it is not uncommon for the person to be perfectly relieved. This medicine is very differently estimated by practitioners: while by many it is most highly extolled, there are not wanting others, who condemn it as both useless and pernicious. Even by some of those who admit its immediate utility, it is dreaded, lest it might eventually produce injury to the constitution. Examples are adduced by Ring, Powell, and Gregory, of its suddenly extinguishing life, by causing apoplexy. But such were rather coincidences than effects, I am inclined to believe, as well from what I have seen myself, as that we are possessed of the most abundant evidence of the older writers, of the safety of the hermodactylus, a very active variety of colchicum, which latter surely cannot be deemed dangerous, if not abused, and, when so displaying the baneful effects they are rather on the primæ viæ and nerves, than on the brain.

Of the thousands of other nostrums, which, at different periods, have been proclaimed, and, for a season, secured more or less complience, the only ones that at present attract my attention, are, Wilson's Tincture, and Reynold's Specific, each probably being composed of colchicum and laudanum, or some other opiate preparation. It may, however, be further deserving of some remark, that several articles either identical with colchicum, as the veratria, or operating very similarly, as digitalis, tobacco, the gratiola, &c. have been employed, and with some repute in the disease. These are the general remedies in regular gout, and which, for the most part, correspondently relieve the local affection. Yet sometimes the agony of the latter is so intense, that the vehement calls for further succour can scarcely be resisted, and I shall proceed to notice the best palliatives.

Much might be expected from opiates, under such circumstances. In the height of the paroxysm, however, it seems now to be sufficiently ascertained, that they have a tendency to aggravate it, by inducing constipation, and increasing the fever, and restlessness. But a course very different was recommended by Brown, and pursued by himself and his disciples. Considering gout to be always connected with debility, he maintained that it should be treated with stimulants, and, among these, that none was more efficacious than opium. That this is mere theory, and refuted by ample experience I need not say. It was the fate of Brown to illustrate, in his own instance, the pernicious nature of his practice. To a large dose of opium, which he took in a paroxysm of podagra that brought on apoplexy, it is said, his death was owing.

Yet, the force of action being abated by proper evacuations, we may then direct opiates with advantage, and, especially, with a diaphoretic, as Dover's powder.

To this limitation of their use, there is an exception. Cases sometimes occur, where, even in the early stage, the pain seems to proceed more from extreme nervous irritation, than proper arthritic inflammation, in which the circulation, though accelerated, is not full or disturbed by the febrile movement; these are benefitted by it.

Leeches afford the most prompt relief to the local affection, and, in contradiction to some writers, may be freely and unhesitatingly directed. These not being to be procured, or, after the use of them, fomentations are to be directed, made of poppy heads, or the hop, or chamomile, or elder flowers. As somewhat analogous, I may also mention the application of a piece of raw fresh meat, a beef steak particularly. Ludricous as this very homely means may seem, I have really known it prove an excellent lenitive. It should, however, be renewed whenever it becomes dry. This, I presume, is a relic of an old custom of soothing wounds and other injuries by putting to them portions of recently killed animals, when still warm. The practice, indeed, in a modified shape, appears to have been continued to our times. Baron Larrey, at least, I am told, was in the habit of enveloping the whole person in the fresh skin of a calf, as a cure for the muscular rigidity caused by cold, as well as a preventive of traumatic tetanus. But to return to our subject. By steaming the inflamed joint, relief is sometimes speedily obtained, and a very convenient machine, for the purpose, is now used. Covering the part with flannel, or cotton, or wool, operates, to a certain extent, in the same way, and, while it assuages suffering, is well calculated to obviate the retrocession of the disease. By Scudamore a mixture is strongly recommended, consisting of three parts of camphorated spirit and one of alcohol, applied warm, from which, however, I have seen no benefit.

Blistering has been proposed, and, since it is so useful in the other phlegmasiæ, with great plausibility of its efficacy.

By Stedman in a treatise on the disease, in which he adopts, as a motto, "Dolor estmedicina doloris," it is indeed, affirmed, that it never fails to do good. Yet it is at present very generally abandoned, and by some, from an apprehension of its tendency to repel the disease internally. We know, however, that sinapisms, and vesicatories are the best means to invite and fasten it down on the extremities, and, I can have no idea of the same remedy producing such opposite effects. My own experience with blisters, in their appropriation to this painful affection, is too limited to decide confidently on the matter. But, from analogy, as well as on account of what I have seen of their operation, I presume that, after a partial reduction of the phlogosis, they would be serviceable; which opinion is strengthened by the consideration, that counter-irritation by some means seems always to have been a favorite practice in such cases. Burning the part with flax, is the suggestion of Hippocrates, with moxa, is adopted among the Chinese; and urtication, in some of the countries of Europe. Deeming counter-irritation of importance, the common epispastics I should prefer.

Every measure of a stimulating or heating character, however, is condemned by some practitioners, and, particularly, by Kinlake, a respectable writer on gout, who maintains that, instead of augmenting the temperature of the part, already too high, by such applications, we should do directly the reverse. His theory of the disease is as fallacious, as the practice deduced from it proves hazardous. Considering the local affection as primary, and the general disturbance of the system to be of a secondary and sympathetic nature, it is inferred, that, by curing the first, the other of course must subside.

That the application of cold to the part, will mitigate the phlogosis, has been shown, from the time of Hippocrates, with whom the practice originated, to the present day. But that it is a perilous expedient, by repelling the disease is equally clear, and is now discarded by discreet practitioners. The only case, in which it is admissible at all, is, where the individual is of sound and vigorous constitution, of which the illustrious discoverer of the circulation, by whom the practice was tried, in regard to himself, ultimately became convinced. Early in life, when robust, he resorted to it, safely and usefully. But, as soon as the feebleness of age began to approach, he was admonished to desist, from the injury he received. Even under the most favourable circumstances, it will be prudent to fortify the stomach, by the previous exhibition of some cordial or stimulant, as a dose of laudanum, or of the carbonate of ammonia, or camphor. An immersion of the feet in cold water, as advised by some, I should think in any case, too hazardous to be ventured. Merely sponging the inflamed part is greatly to be preferred. Covering it with cabbage leaves, and still more with those of the tulip poplar, I have known to be productive of ease. They operate, however, differently, the one being wilted, as a fomentation, and the other in the natural state, by lowering temperature and occasioning the most copious dewy-like exhalation.

The course of treatment, now detailed, has reference mainly to the more violent states of gout. It often, however, assumes a mitigated shape, or appears in the aged or otherwise infirm, and, here, the measures are to be tempered accordingly.

On the subsidence of the paroxysm, little is commonly required to be done, where the integrity of the constitution is preserved. Moderately nutritious diet, with a glass or two daily, of pure Madeira or Sherry wine, and some attention to the bowels, are all that is actually exacted to secure a perfect convalescence. The joints, however, may continue swollen and stiff, to remove which, frictions with a stimulating liniment, and a flannel roller, when edematous, are found most effectual.

Concerning chronic gout, in its more inveterate states, not much can be effected towards a cure, and, for the most part, our efforts are limited merely to a palliation of urgent affections. The remedies here are nearly the same, as in the acute disease, though none of them can be urged to any extent. As the management, on the whole, is very similar to that of some of the forms of atonic gout, I shall postpone the details of it, till I arrive at the consideration of that division of the subject. In many instances, the animal machine becomes so broken, and out of order, by the ravages of the disease, that no skill can repair it. But, when less injured, some encouragement is afforded, and endeavors should be made towards its rectification. As preliminary to any such attempt, the entire economy is carefully to be surveyed, with a view to the detection of the principal lesions. Nearly always, it will be found, that the abdominal viscera, are materially in fault, the stomach, bowels and liver especially. Great advantages are sometimes derived, in such cases, from the cautious use of the blue pill and the taraxicum mixture, to be followed ultimately, by a course of the mildest preparations of steel, and a properly regulated regimen. As pains, nervous inquietudes, and the want of sleep, are pretty uniform attendants on this condition, worrying, and exhausting in their effects, these must be mitigated by lenitives, among the best of which, is henbane, or an opiate in combination with a gentle diaphoretic, at night. Confidence is however reposed by some, at this conjuncture, in the compound syrup of sarsaparilla, volatile tincture of guiacum, the terebinthinates, the savin, and similar articles. But, so far as I have seen, these are very equivocal remedies, and ordinarily prove so offensive to the stomach, that they are at once rejected, or cannot be continued to any efficient end. The savin, however, when tolerated for a considerable length of time, I have known, in several instances, to remove the extraneous matter in the joints, and thereby completely to restore freedom in their movements. This is the most useful property of the article in its application to gout. But, in relation to the articular derangements, I prefer treating of them fully under the head of chronic rheumatism, in which they more frequently occur. I have now only to add, that, whenever practicable, individuals with chronic gout, should make a fair trial of the thermal springs of Virginia, which often do more than all other means.

